# Safety and Efficacy of Low-Dose Versus High-Dose Parenteral Ketorolac for Acute Pain Relief in Patients 65 Years and Older in the Emergency Department

**DOI:** 10.7759/cureus.40333

**Published:** 2023-06-12

**Authors:** Emma Platt, Jessica M Neidhardt, Bradley End, Courtney Cundiff, Wei Fang, Vivian Kum, Heather Tucker, Jeffrey M Quedado

**Affiliations:** 1 Pharmacy, West Virginia University, Morgantown, USA; 2 Emergency Medicine and Medical Education and Simulation, West Virginia University, Morgantown, USA; 3 Emergency Medicine and Medical Education, West Virginia University, Morgantown, USA; 4 Statistics, West Virginia University, Morgantown, USA; 5 Pharmacy, NewYork-Presbyterian Hospital, New York, USA; 6 Emergency Medicine, West Virginia University, Morgantown, USA; 7 Pharmacology and Therapeutics, West Virginia University, Morgantown, USA

**Keywords:** nonopioid analgesia, nsaids, geriatrics, non-opiate pain control, ketorolac

## Abstract

Background

There is limited data surrounding acute pain management in elderly ED patients. Ketorolac is a potent non-steroidal anti-inflammatory drug (NSAID) with dose/duration-dependent side effects. There is evidence that an analgesic ceiling effect exists for parenteral ketorolac doses greater than 10 milligrams (mg); however, this has not been studied in patients 65 years and older.

Methods

This was a retrospective chart review of ED patients 65 years and older who received at least one dose of parenteral ketorolac. Patients were separated into two cohorts based on the ketorolac dose received: 15 mg IV or 30 mg intramuscular (IM) and 30 mg IV or 60 mg IM. The primary objective was to evaluate the analgesic efficacy of parenteral ketorolac doses measured as needing rescue analgesia from 30 minutes to 2 hours after ketorolac administration. Secondary objectives included changes in pain scores and the occurrence of adverse drug events commonly associated with ketorolac.

Results

Two-hundred and sixty patients received ketorolac doses of 15 mg IV or 30 mg IM, and 52 received 30 mg IV or 60 mg IM. The primary outcome occurred in seven of 52 patients who received ketorolac 30 mg IV or 60 mg IM and 17 of 260 patients who received ketorolac 15 mg IV or 30 mg IM (13.5% vs. 6.5%, p=0094; OR: 2.22, 95% CI: 0.87-5.67). The average change in pain scores were 2.9 (±3.1) and 2.8 (±2.9) for patients who received doses 30 mg IV or 60 mg IM compared to doses 15 mg IV or 30 mg IM, respectively (p=0.154). The occurrence of adverse events was low in both groups.

Conclusion

Parenteral ketorolac doses of 15 mg IV or 30 mg IM did not demonstrate a greater need for rescue analgesia compared to doses of 30 mg IV or 60 mg IM.

## Introduction

As life expectancy continues to rise, the population of patients 65 years and older continues to grow and is expected to double by 2060 [1]. Generally, geriatric patients are more likely to be hospitalized, accounting for roughly 20% of ED visits [2]. Physiological changes, comorbidities, and polypharmacy increase the potential for adverse drug events and can complicate medication selection when treating these patients in the ED [2-3]. This is especially true in treating acute pain, where many agents, such as non-steroidal anti-inflammatory drugs (NSAIDs) and opioids, should be avoided or used with caution, according to the American Geriatric Society Beers Criteria [4]. Ketorolac tromethamine is a parenteral NSAID that may be given intravenously (IV) or intramuscularly (IM) [5]. It provides potent analgesic and anti-inflammatory effects and is indicated for the short-term management of moderate-to-severe acute pain that requires analgesia at the opioid level [5]. However, ketorolac is also associated with adverse GI, renal, and cardiovascular events, many of which demonstrate a dose-dependent and/or duration-dependent effect [5]. Anderson GL et al. found that using a single dose of IV ketorolac in ED patients 65 years and older was not associated with increased adverse events [6]. Ketorolac may also demonstrate an analgesic ceiling effect, where higher doses may not provide additional benefit. In a study by Motov S et al., IV ketorolac doses of 10, 15, and 30 mg provided similar pain reduction between groups [7]. Similarly, a study by Turner NJ et al. found that a 15 mg IM dose of ketorolac was non-inferior to a 60 mg IM dose for short-term pain relief of acute musculoskeletal pain [8]. However, these studies only included patients 18-65 years. While pain management can be difficult in all patients, those 65 years and older tend to be particularly challenging. The elderly population is more susceptible to adverse events, which can limit provider options when selecting analgesic therapy. Given that the adverse effects associated with ketorolac are both dose and duration-dependent, lower doses of parenteral ketorolac may have greater utility in acute pain management in elderly patients.

Importance

There is limited data surrounding pain management in elderly ED patients. The potential analgesic ceiling effect with parenteral ketorolac has not been investigated in patients 65 years and older. The results from this study lend support to the growing body of evidence that IV ketorolac doses greater than 15 mg IV or 30 mg IM may be unnecessary, especially in one of our most vulnerable populations.

Goals of this investigation

The goal of this study is to assess if 15 mg IV or 30 mg IM of parenteral ketorolac (low-dose ketorolac [LDK]) provides similar pain reduction as 30 mg IV or 60 mg IM of parenteral ketorolac (high-dose ketorolac [HDK]) in ED patients 65 years and older. 

## Materials and methods

Study design and setting

This was a single health-system, retrospective study of ED patients 65 years and older who received at least one dose of parenteral ketorolac. Data were obtained from the West Virginia University (WVU) Medicine electronic medical record (EMR). The local IRB approved the study, with the IRB protocol number 2111454746. 

Selection of participants

Patients were included if they were 65 years or older and had received at least one dose of parenteral ketorolac in the ED between August 1, 2018, and July 31, 2021. Exclusion criteria were patients that were less than 65 years of age, those that did not receive IM/IV ketorolac during the ED encounter, or those that did not have a pain assessment and/or pain response documented in the medical record. Six-hundred and sixty-five patients were identified, of which 312 were included in the analysis. Fifty-two of these patients comprised the cohort of patients who received HDK. Two-hundred and sixty patients were randomly selected from the remaining population to serve as the cohort of patients who received LDK. 

Outcomes

The primary outcome was analgesic efficacy measured as needing rescue analgesia within 30 minutes to 2 hours after ketorolac administration, as documented in the patient’s EMR. Secondary outcomes included the change in pain score after ketorolac administration and the incidence of adverse drug events commonly associated with ketorolac within 72 hours of administration. Pain scores were measured using a numeric scale ranging from 0 to 10, with larger numbers representing greater pain. Reported adverse events included GI effects (bleeding, peptic ulcer nausea, vomiting, and abdominal pain), nephrotoxic effects (acute kidney injury [AKI]: defined as an increase in serum creatinine of 0.3 mg/dL or more within 48 hours), cardiovascular effects (edema or thrombotic events), and central nervous system (CNS) effects (dizziness, headache, confusion, agitation, or hallucinations).

Statistical analysis

Categorical data were expressed as percentages, and continuous data as means with SDs. To compare baseline characteristics between groups, a Wilcoxon rank sum test was used for continuous variables and a Chi-square test for categorical variables. The primary outcome of the need for rescue analgesia was evaluated using a Chi-square test and a univariate logistic regression. Additionally, a multivariable logistic regression analysis was performed to assess the association between the primary outcome and dose of ketorolac, controlling for confounding effects from baseline pain scores and concomitant analgesia. Results from the logistic regression analyses are reported as odds ratios (95% CI), the Wilcoxon rank sum test result is reported as a p-value, and the significance level was set at 0.05. Incidences of adverse drug events are reported with descriptive statistics.

## Results

Characteristics of study subjects

Six-hundred and sixty-five patients were identified, of which 312 were included in the analysis. Fifty-two patients were sorted into the group who received HDK (30 mg IV [n = 50] or 60 mg IM [n=2]), and 260 were included in the group who received LDK (15 mg IV [n=227] or 30 mg IM [n=33]) (Table 2). Baseline demographics were relatively balanced between groups with a few notable differences. Patients who received LDK were slightly older than those who received HDK, and those who received HDK were more likely to be admitted to the hospital than those who received LDK (Table 1). Regarding medication characteristics, a greater proportion of patients in the HDK group received analgesia more than 30 minutes prior to the administration of ketorolac, but concomitant analgesia was similar between groups (Table 2). The availability of baseline pain scores was similar between both groups; however, more patients in the HDK group had available pain scores after using ketorolac (Table 3). The average baseline pain score was slightly higher in the HDK group (Table 3). Of note, there was less than 1 point of difference between groups. Additionally, the average pain score after ketorolac administration was similar between groups (Table 3).

**Table 1:** Baseline demographics. ^a ^CAD defined as stroke, myocardial infarction, or peripheral artery disease ^b ^Other sources of pain included face, mouth, chest, and eye eGFR: Estimated glomerular filtration rate; CAD: Coronary artery disease; CHF: Congestive heart failure; DAPT: Dual antiplatelet therapy.

Demographic	Ketorolac 30 mg IV or 60 mg IM (n=52)	Ketorolac 15 mg IV or 30 mg IM (n=260)	P-value
				Age (years) - avg. (SD)	70 (5)	73 (7)	0.003
Female sex - no. (%)	24 (46)	155 (60)	0.091
Weight <50 kg - no. (%)	1 (2)	10 (4)	0.698
Renal function - no. (%)			
eGFR > 50 mL/min	44 (85)	189 (73)	0.081
eGFR 30-50 mL/min	4 (8)	31 (12)	0.476
eGFR < 30 mL/min	-	2 (1)	1.000
eGFR unavailable	4 (8)	38 (15)	0.045
Admission - no. (%)	22 (42)	71 (27)	0.045
History of GI bleed - no. (%)	4 (8)	18 (7)	0.771
CAD ^a^ - no. (%)	16 (31)	88 (34)	0.748
CHF - no. (%)	3 (6)	23 (9)	0.591
Anticoagulation use - no (%)	2 (4)	33 (13)	0.200
Antiplatelet use - no (%)	45 (87)	204 (78)	0.185
DAPT	22 (42)	98 (38)	0.532
Monotherapy	23 (44)	106 (41)	0.644
Source of pain - no (%)			
Back	12 (23)	49 (19)	0.483
Abdominal	7 (14)	41 (16)	0.356
Flank	5 (10)	16 (6)	0.363
Extremities	8 (15)	49 (19)	0.555
Head	9 (17)	31 (12)	0.289
Other ^b^	11 (21)	74 (28)	0.280

**Table 2:** Medication characteristics. ^a ^Analgesia prior to ketorolac defined as given greater than 30 minutes before ketorolac. ^b ^Concomitant analgesia defined as analgesia administered within 30 minutes of ketorolac. IM: Intramuscular.

Characteristic	Ketorolac 30 mg IV or 60 mg IM (n=52)	Ketorolac 15 mg IV or 30 mg IM (n=260)	P-value
				IM administration - no. (%)	2 (4)	33 (13)	-
Analgesia prior to ketorolac ^a^ - no. (%)	20 (38)	58 (22)	002
Opioid	19 (83)	42 (66)	-
Non-opioid	4 (17)	22 (34)	-
Concomitant analgesia ^b^ - no. (%)	12 (23)	64 (25)	1.00
Opioid	4 (29)	19 (28)	-
Non-opioid	10 (71)	50 (72)	-
Rescue analgesia - no. (%)	7 (13.5)	17 (6.5)	0.094
Opioid	4 (57)	9 (53)	-
Non-opioid	3 (43)	8 (47)	-
Analgesic Prescription - no. (%)	17 (33)	70 (27)	0.40
Opioid	11 (42)	37 (37)	-
Non-opioid	15 (58)	63 (63)	-

**Table 3:** Pain scores.

Pain score	Ketorolac 30 mg IV or 60 mg IM (n=52)	Ketorolac 15 mg IV or 30 mg IM (n=260)	P-value
				Baseline available - no. (%)	44 (85)	216 (83)	1.000
Baseline value - avg. (SD)	7.9 (1.7)	7.14 (2.4)	0.037
Post ketorolac available - no. (%)	22 (42)	73 (28)	0.048
Post ketorolac value - avg. (SD)	5.0 (3.5)	5.4 (3.3)	0.410
Change in pain score - avg. (SD)	2.9 (3.1)	2.8 (2.9)	0.154

Primary outcomes

Need for rescue analgesia was not statistically significant between groups, with 13.5% (n=7) in the HDK group and 6.5% (n=17) in the LDK group; OR 2.22 (95% CI: 0.87-5.67; p=0.094) (Table 4). A further analysis was conducted to determine if the need for rescue analgesia differed by the presence of concomitant analgesia or baseline pain score. No significant difference was found when accounting for these factors; OR 2.07 (95% CI: 0.79-5.39; p=0.138) (Table 4).seline pain score. No significant difference was found when accounting for these factors; OR 2.07 (95% CI 0.79-5.39; p=0.138) (Table 4).

**Table 4:** Analgesic efficacy. ^a^ Univariate logistic regression only looking at ketorolac dose. ^b ^Multivariable logistic regression controlling for concomitant analgesia and baseline pain score.

Outcome	Ketorolac 30 mg IV or 60 mg IM (n=52)	Ketorolac 15 mg IV or 30 mg IM (n=260)	OR (95%Cl)	P-value
					Rescue analgesia - no. (%)			2.22	p=0.094
Univariate ^a^	7 (13.5)	17 (6.5)	(0.87-5.67)						
Rescue analgesia - no. (%)			2.07	p=0.138
Multivariable ^b^	-	-	(0.79-5.39)	
Change in pain score - avg (SD)	2.9 (3.1)	2.8 (2.9)	-	p=0.154

Secondary outcomes

TThe change in pain score was not statistically significant between groups, with a 2.9 (±3.1) reduction in the HDK group and a 2.8 (±2.9) reduction in the LDK group (p=0.154) (Table 4). Overall, adverse drug events were rare in both groups. Edema, determined by the need for diuretic therapy, was the most common potential adverse drug event reported, with 4% (n=2) in the HDK cohort and 3% (n=9) in the LDK cohort (Table 5).

**Table 5:** Adverse drug events.

Adverse event	Ketorolac 30 mg IV or 60 mg IM (n=52)	Ketorolac 15 mg IV or 30 mg IM (n=260)
			Gastrointestinal - no. (%)	-	-
Acute Kidney Injury (AKI) - no (%)	-	5 (2)
Cardiovascular - no. (%)		
Thrombotic event	-	1 (<1)
Edema	2 (4)	9 (3)
Central Nervous System (CNS) - no. (%)	1 (<1)	1 (<1)

## Discussion

There was no statistical difference between patients in the LDK group compared to the HDK group for the primary outcome of analgesic efficacy measured as a need for rescue analgesia. Additionally, the change in pain scores between groups was not statistically different. These results add to the growing body of evidence that ketorolac may exhibit an analgesic ceiling effect [7-8]. Given the dose and duration-dependent side effect profile of ketorolac, the ability to provide adequate analgesia at lower doses is desirable. Previous studies evaluating this theory have excluded patients 65 years and older because doses greater than 15 mg IV or 30 mg IM are typically not recommended [7-8]. However, these recommendations are based solely on reducing adverse drug events. The findings from this study further reinforce the use of ketorolac doses of 15 mg IV or 30 mg IM or less in patients 65 years and older by granting physicians the confidence to provide adequate analgesia. While the retrospective design of this study opens it up to significant weaknesses, a randomized control trial would not be ethically feasible since ketorolac doses greater than 15 mg IV or 30 mg IM are not recommended in patients 65 years and older [5]. The overall low number of reported adverse drug events between groups likely resulted from utilizing an ED population with limited visits (often <24 hours) and limited follow-up. For the adverse events that were reported, there were no significant differences between groups; however, these results should be interpreted in the context of limited data. No patient in this study received additional doses of ketorolac after the initial dose.

Limitations

This study has several important limitations. As mentioned previously, the retrospective chart review design opens it up to areas for improvement, such as a dependence on the accuracy of EMR documentation, missing data points, and an inability to control for confounding variables. The accuracy of documentation may further be reduced in the environment of an ED, given the high acuity of patients and potentially hectic, heavy workload. Similarly, this may also contribute to a greater degree of undocumented information, such as in the case of the relatively low number of available pain scores after the administration of ketorolac. While a logistic regression analysis was utilized to account for confounding variables such as concomitant analgesia and baseline pain score, many factors may influence analgesic efficacy that this study could not assess. Additionally, the clinically accepted practice of a multimodal analgesia approach to pain management makes it difficult to determine the true analgesic efficacy of ketorolac alone. Because pain is a subjective finding, there is room for a great deal of interpatient variability. Markers of inflammation using higher doses of ketorolac were not measured as an outcome. Lastly, the utilization of an ED population made it difficult to detect adverse drug events due to the relatively short visit time and limited follow-up data.

## Conclusions

Ketorolac doses of 15 mg IV or 30 mg IM did not demonstrate a greater need for rescue analgesia than doses of 30 mg IV or 60 mg IM, nor did they result in a greater pain score reduction. These results extend the theory of a ceiling dose effect with ketorolac to patients 65 years and older. Using parenteral ketorolac doses of 15 mg IV and 30 mg IM may give physicians a higher level of confidence in providing adequate analgesia while reducing the risk of adverse events when treating acute pain in elderly patients.
